# The Impact of Climate on Human Dengue Infections in the Caribbean

**DOI:** 10.3390/pathogens13090756

**Published:** 2024-09-03

**Authors:** Kirk Osmond Douglas, Karl Payne, Gilberto Sabino-Santos, Peter Chami, Troy Lorde

**Affiliations:** 1 Centre for Biosecurity Studies, The University of the West Indies, Cave Hill Campus, Cave Hill, Bridgetown BB11000, Barbados; 2 Centre for Environmental Resource Management, The University of the West Indies, Cave Hill Campus, Cave Hill, Bridgetown BB11000, Barbados; karl.payne@cavehill.uwi.edu; 3 Department of Microbiology and Immunology, Tulane University School of Medicine, 1430 Tulane Ave Rm. 5718, New Orleans, LA 70112, USA; gsabino@tulane.edu; 4 Centre for Virology Research, School of Medicine in Ribeirao Preto, University of Sao Paulo, 3900 Bandeirantes Ave, Ribeirao Preto 14049-900, SP, Brazil; 5 Department of Computer Science, Mathematics, & Physics, The University of the West Indies, Cave Hill Campus, Cave Hill, Bridgetown BB11000, Barbados; peter.chami@cavehill.uwi.edu; 6 Department of Economics, The University of the West Indies, Cave Hill Campus, Cave Hill, Bridgetown BB11000, Barbados; troy.lorde@cavehill.uwi.edu

**Keywords:** climate change, dengue, tropics, Caribbean, public health, biosecurity, rainfall, temperature, infectious diseases, vector-borne

## Abstract

Climate change is no longer a hypothetical problem in the Caribbean but a new reality to which regional public health systems must adapt. One of its significant impacts is the increased transmission of infectious diseases, such as dengue fever, which is endemic in the region, and the presence of the *Aedes aegypti* mosquito vector responsible for transmitting the disease. (1) Methods: To assess the association between climatic factors and human dengue virus infections in the Caribbean, we conducted a systematic review of published studies on MEDLINE and Web of Science databases according to Preferred Reporting Items for Systematic Reviews and Meta-Analyses (PRISMA) criteria. (2) Results: In total, 153 papers were identified, with 27 studies selected that met the inclusion criteria ranging from the northern and southern Caribbean. Rainfall/precipitation and vapor pressure had a strong positive association with dengue incidence, whereas the evidence for the impact of temperatures was mixed. (3) Conclusions: The interaction between climate and human dengue disease in the Caribbean is complex and influenced by multiple factors, including waste management, infrastructure risks, land use changes, and challenged public health systems. Thus, more detailed research is necessary to understand the complexity of dengue within the wider Caribbean and achieve better dengue disease management.

## 1. Introduction

Dengue fever (DF) is the foremost human arboviral disease worldwide in tropical and subtropical regions. Dengue virus (DENV) is a global health threat responsible for over 100 million infections annually and primarily acquired through the bites of infected mosquitoes [1]. Natural DENV transmission involves the feeding of infected female mosquitoes on a susceptible vertebrate host, generally a human (urban cycle) or non-human primate (sylvatic cycle) [2]. DF is an emerging infectious disease threat in the Caribbean and the Americas. DENV infection causes DF, severe dengue (SD), dengue hemorrhagic fever (DHF), or dengue shock syndrome (DSS). It is estimated that >100 million DENV infections occur annually around the globe [3]. Dengue has spread rapidly since the 1960s and continues to spread with 30–54.7% (~2–3 billion people) of the world’s population in countries where DENV is transmissible [4,5]. Dengue is endemic throughout the Caribbean, and DF bears similar clinical symptomology to other endemic infectious diseases, including hantavirus, Chikungunya virus, Mayaro virus, Zika virus, and *Leptospira* infections.

A significant increase in dengue cases has occurred within the Caribbean and the Americas. The steep rise in cases was noted by the Pan American Health Organization (PAHO), where in 2024, almost three times as many cases were reported from the Americas than for the same period 1 year prior [6]. Warmer temperatures and higher rainfall were reportedly creating ideal conditions for the *Aedes aegypti* mosquito, which is the primary vector of dengue. Specifically in Barbados, there has been a major outbreak of dengue in Barbados since October 2023. As of April 2024, a total number of 2915 clinically suspected dengue cases and 1059 laboratory-confirmed cases were recorded in Barbados [7]. The main DENV serotype affecting Barbados during this period was type 2, as well as type 3.

DENV is an arbovirus with a single-strand positive-sense ribonucleic acid (RNA) genome (approximately 10,700 bases), surrounded by a nucleocapsid enclosed with a lipid envelope [8]. The DENV genome comprises a single open reading frame (ORF), which co- and post-translationally cleaves into three structural (capsid [C], pre-membrane [prM], and envelope [E]) and seven non-structural (NS1, NS2A, NS2B, NS3, NS4A, NS4B, and NS5) proteins [8]. DENV is transmitted by mosquitoes, mainly *Aedes aegypti* and *Aedes albopictus*. Mosquitoes, generally *Aedes aegypti*, become infected by biting an infected host during the viremic period of infection. The virus passes from the infected host to the mosquito’s digestive tract, where it replicates during the extrinsic incubation period, characteristically 10–14 days and high ambient temperatures [9]. After the extrinsic incubation period, DENV is disseminated through the hemolymph to replicate in the fat body, trachea, and salivary glands of the mosquito [9]. Infected mosquitoes continue to transmit DENV through biting until death, and DENV infection alters mosquito behavior, causing them to increase their biting frequency [10].

Small island developing states (SIDSs), like those in the Caribbean, are projected to experience multiple and compound climate-related risks [11]. Land limits to adaptation will be reached for several key impacts, resulting in residual impacts, particularly on human health. Climatic events such as flooding, droughts, heat waves, and sea level rise have negatively impacted climate-sensitive health problems such as chronic non-communicable diseases (NCDs) and communicable disease morbidity and mortality [12]. This includes certain vector-, food-, and water-borne diseases like dengue, hantavirus, and leptospirosis [13]. This makes climate change critical and pivotal to other human health threats, potentially amplifying DENV infection risk and other negative impacts.

Previous Intergovernmental Panel on Climate Change (IPCC) reports indicate that human health in SIDSs can be seriously compromised by a lack of access to adequate and safe freshwater and nutrition [14,15]. There is also a growing concern in Caribbean SIDSs that freshwater scarcity and more intense droughts, hurricanes, and tropical storms could lead to a deterioration in standards of sanitation and hygiene [16]. In such circumstances, increased exposure to a range of health risks, including communicable diseases, would be a distinct possibility. This is particularly concerning with rainwater harvesting and poor water storage practices that enable mosquito breeding in the Caribbean [17]. Climate projections for the Caribbean indicate that the region will experience higher temperatures, more intense rainfall activity, and more frequent and intensive weather systems [18]. This signals that future climate variability is imminent, and concomitant with this are the increased risks of infectious disease outbreaks, in particular, endemic mosquito-borne diseases such as DF, Zika fever, Mayaro fever, and Chikungunya fever, and the potential for a resurgence of malaria in the Caribbean [19,20,21,22,23]. Climatic factors, including rainfall, humidity, and temperature, increase the risk for mosquito vector growth and proliferation and, thus, an increased chance of DENV infections, as evidenced by correlating these factors with dengue cases [24]. These factors enhance the growth rate of vectors transmitting DENV and increase the risk of human-infected mosquito contact and virus transmission.

El Niño refers to the warming of sea surface temperatures in the central and eastern equatorial Pacific Ocean [25]. This warming contributes to major changes in weather patterns throughout the globe. Globally, El Niño-intensified rainfall leads to higher temperatures in the North Atlantic, supporting conditions advantageous for mosquito breeding and increased risk of mosquito-borne diseases [26,27]. Therefore, robust public health practices, including surveillance and monitoring of mosquito activity, as well as education, would be required during El Niño periods. This would reduce the observed adverse consequences such as dengue outbreaks and epidemics [26]. Early warning systems (EWSs) can also improve the actions of public health agencies via modeling climate-infectious disease dynamics to improve disease outbreaks and epidemic planning [28].

To understand the influence of various climatic factors on human DENV infections in the Caribbean, we conducted a systematic review to analyze the bibliographic evidence from peer-reviewed studies conducted in the Caribbean. This is the first ever systematic review conducted for human dengue virus infections and climate in the Caribbean. Given the climate predictions for the Caribbean, it is prudent to grasp the present and future influences of climate change on human DENV infection dynamics in this region.

## 2. Methods

### 2.1. Bibliographic Search

**Figure 1:**
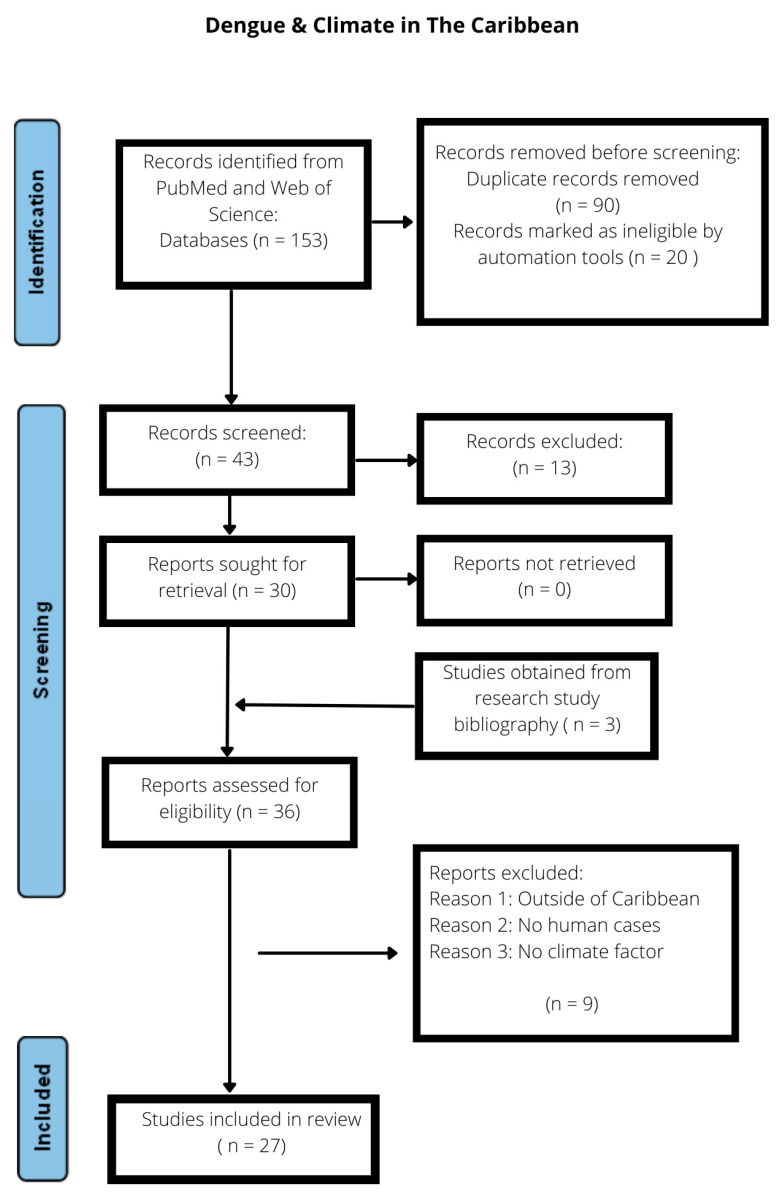
PRISMA bibliographic search (MEDLINE and Web of Science), screening, and study selection flowchart.

Using a previous method based on population, exposure, and outcome (PECOS) criteria, we performed a bibliographic search for all relevant peer-reviewed journal publications on climate and DENV infections in the Caribbean using a pre-established search strategy (Table 1 and Figure 1) [29,30]. For this systematic review, we defined the population of interest as humans of all ages and sexes, the exposure as short- and long-term climate variability, and the outcome as SD of DHF/DSS, NSD, DF, or serologically or reverse transcriptase polymerase chain reaction (RT-PCR) confirmed human DENV infections.

### 2.2. Selection Criteria

For the defined inclusion criteria, only human DENV infections were considered (with no limits on age or sex), exposures were climate variability over the short and long term (a minimum of 2 years of monitoring), and finally, the outcome was acute DENV infections with limitations to studies conducted within the wider Caribbean. We defined the wider Caribbean as the Caribbean Community (CARICOM) member states and all other island territory states in the Caribbean Sea, including Suriname, Belize, Cayman Islands, Aruba, Bonaire, Curaçao, Saint Marteen, Martinique, Cuba, Turks and Caicos, Puerto Rico, U.S. and British Virgin Islands, Anguilla, Saint Barthélemy, Saint Eustatius, Saba, Suriname, and French Guiana. The rationale for this is that these are all geographically similar, involved in vibrant trade and travel, and likely to be affected by climatic conditions similarly. This 2-year minimum was established to mitigate against the impact of seasonality and short-term weather changes. The exclusion criteria included (a) non-primary research studies, (b) studies outside of the Caribbean region, and (c) studies investigating only DENV infections in mosquito vectors and associations with climate factors but no human DENV infections. Studies investigating the association of climatic factor variability and acute DENV infections and or disease in the Caribbean were all included for collection and analysis. All dengue cases, including probable and confirmed cases, were diagnosed per the case definitions as per the 1997 WHO dengue guidelines for 2008, and a DF and severe dengue (SD) case were defined as per the 2009 WHO dengue guidelines for 2009–2016.

The exclusion criteria for the systematic review included (a) non-primary research studies, (b) studies outside of the Caribbean and broader CARICOM region, and (c) studies investigating only DENV infections in animal or vector hosts and associations with climate factors but not with human DENV infections. Studies investigating the association of climatic factors and acute human DENV infections and disease in the wider CARICOM region and the Caribbean were all included for collection and analysis.

### 2.3. Search String

A suitable search string was developed, including the search terms framed by the relevant inclusion and exclusion criteria [31]. This search string was designed as follows (climate “OR” weather “OR” drought “OR” season* “OR” rain* “OR” precipitation AND human AND dengue “OR” dengue haemorrhagic fever “OR” DHF “OR” severe dengue “OR” SD “OR” dengue virus “OR” DENV AND Caribbean), similar to a previous study [30]. Adaptations were made for other database formats. Notably, this bibliographic search lists studies in these databases updated as of 27 June 2023. The relevant studies matching the inclusion criteria that were published after this time were included in the Discussion. In addition, using relevant review journal articles on climate and human DENV infections in the Caribbean, additional studies meeting the inclusion criteria were added to the list of selected studies. Searches were conducted on MEDLINE using PubMed and Web of Science databases. Notably, this bibliographic search lists studies in these databases updated as of 27 June 2023.

### 2.4. Study Selection and Quality Assessment

Two independent reviewers (G.S., K.P.) selected the studies for which full texts were obtained, and the two readers (P.C., T.L.) then decided independently about the final inclusion of articles. In cases of disagreement, an arbiter (T.L.) was involved. In addition, using relevant review journal articles on climate and human dengue infections, additional studies meeting the inclusion criteria were added to the list of selected studies. Each methodology of the selected studies was analyzed for quality, including the risk of bias, using the Scottish Intercollegiate Guidelines Network (SIGN) and Critical Appraisal Skills Programme (CASP). The outcome measurement was included in the checklist with a higher rating for serologically confirmed dengue cases in preference to self-reported or symptomatic diagnoses. The influence of confounders was also accounted for, as previously done [30]. Assessments were conducted and recorded for each selected study using a 3-point scale prescribed by SIGN, which indicates ++ as very high quality, + as high quality, and − as low quality. This quality rating followed the range of fulfillment of the criteria regarding the validity of results. The complete checklist is shown in Appendix A.

Data from these selected studies were extracted independently to compile an evidence table (Table 2), which provided pertinent information on study location, number of human cases, study period, exposure, outcome, and notable results related to climate and related co-factors. In addition, metrics and statistical assessments used to assess the models and analyses and the assigned quality of each study were noted (Table 3). Given the varied nature of methodologies utilized in the selected studies and varied geographical locations, a qualitative synthesis of study data was conducted rather than a quantitative analysis performed for meta-analysis. Other associated co-factors included other social, demographic, socioeconomic, geospatial, and biotic factors.

For human DENV infections, a serological and or molecular test confirmation of infection was the outcome, and the effect measure was variable given the heterogeneity of the study methods used in the selected studies. For missing summary statistics, the evaluation of the study design, outcome, and results were used to deduce the vigor of the study under examination to determine aptness and accuracy.

## 3. Results

### 3.1. Bibliographic Search

To facilitate this systematic review, a search of the peer-reviewed literature was conducted following Preferred Reporting Items for Systematic Reviews and Meta-Analyses (PRISMA) checklist guidelines among the relevant MEDLINE (via PubMed) and Web of Science databases (Appendix A). This resulted in the collection of 111 studies primarily retrieved via MEDLINE via PubMed and 42 findings using the Web of Science database. After the application of the relevant inclusion criteria, a total of 27 studies were selected, which included studies involving (a) human DENV infections only, available as full-text articles, with relevant abstracts or study titles (with climate and human DENV infections), and (b) conducting analysis of climate variables including temperature, rainfall, vapor pressure (VP), humidity, mean sea level and seasonality and dengue cases and or incidence, and (c) countries from with the Caribbean and wider CARICOM region (geographical limitation) (Table 1 and Figure 1).

### 3.2. Selected Studies

**Figure 2:**
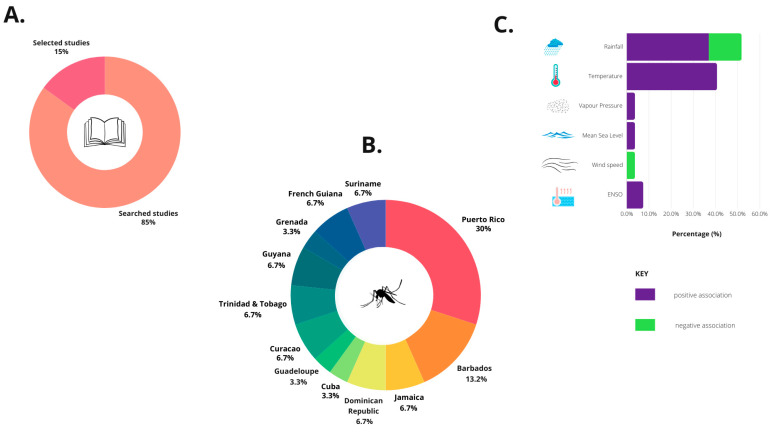
(**A**) The percentages of peer-reviewed studies examining the association of climate variables and human dengue infections following bibliographic searches from MEDLINE and Web of Science databases. (**B**) The percentage composition of selected studies of the association of climate variables and human DENV infections in the Caribbean region by country. (**C**) The percentages of selected studies with associations of specific climate variables and human DENV infections in the Caribbean region.

There were 27 selected studies for this review investigating the association of climatic factors with human DENV infections in the CARICOM and territories in the Caribbean Sea region, including 13 different English-, Spanish-, French-, and Dutch-speaking countries (Figure 2B and Table 2). Each selected study was analyzed to determine the association of climatic variables with human DENV infections. Rainfall was the climatic factor or exposure most associated with dengue infections approximately 51.9% (14/27) of selected studies, while the temperature was associated with 40.7% (11/27) of these studies, followed by ENSO 7.4% (2/27), humidity 3.7% (1/27) [32], wind speed 3.7% [33], and vapor pressure 3.7% (1/27) [33] (Figure 2 and Table 2). Rainfall was negatively associated with dengue cases in 14.8% (4/27) of the selected studies [34,35,36,37] and positively associated with dengue cases in 37% (10/27) of the selected studies [38,39,40,41,42,43,44,45,46] (Figure 2 and Table 2). Temperature was positively associated with dengue cases in 40.7% (11/27) of the selected studies [32,33,39,40,47,48,49,50,51,52,53] (Figure 2). The proportion of studies represented here refers to the proportion of studies that included these indicators and found them to be associated with dengue incidence. Sunshine hours and other different climate factors were not investigated in any of the selected studies. Other related co-factors assessed included energy change [53], poverty index [39], forested/scrubland habitats [41], and susceptibles index [42] (Figure 2 and Table 2).

**Table 2:** Analysis of selected published peer-reviewed studies.

Study	Quality	Study Location	Study Design	Time Period	Climatic Variables	Outcome	CO-FACTORS	Statistical Methods	Results
Keating et al., 2001 [52]	+	Puerto Rico	Multivariate linear regression	1988–1992	Seasonal temperatures	Dengue cases	None	Regression analysis and Durbin–Watson test	Temperature has a positive effect on dengue cases reported each month with a lag of 12 weeks or 3 months. Other factors may be influencing seasonal dengue incidence.
Schreiber et al., 2001 [53]	+		Multivariate stochastic models	1988–1993	Temperature, rainfall	Dengue cases	None	Pearson’s product–moment correlation coefficient	The mean seasonal variation in dengue is highly related (*R*^2^ = 88.1%) to the mean seasonal climate variation, with those thermal and energy variables immediately preceding the dengue response showing the strongest relationships. However, moisture variables, predominantly in the form of surplus, are more influential many weeks in advance. For the interannual model (*R*^2^ = 44.1%), energy change, thermal change, and moisture variables are significant across the 8-week period, with moisture variables playing a stronger role than in the intra-annual model.
Jury, 2008 [38]	+		Bayesian model	1979–2005	Rainfall, wind speed, temperature, and air pressure	Dengue cases	None	K function analysis and Barton David Knox	A positive association of rainfall with dengue was observed with no appreciable lag time. While temperature was positively associated with year-to-year variability of dengue cases.
Johansson et al., 2009a [39]	++		DLM	1986–2006	Temperature and rainfall	Dengue cases	Social vulnerability (household income and % persons below poverty line)	95% CI	Temperature influences dengue incidence in cooler mountain regions. Rainfall’s strongest influence is in the dry southwestern coast. Areas with higher poverty index had more dengue cases.
Johansson et al., 2009b [40]	++		Wavelet analysis	1993–2016	Temperature, precipitation, and ENSO index	Dengue cases	None	Monte Carlo test of duration	Temperature and rainfall strongly coherent with dengue with 1 y periodicity. A strong link with ENSO and dengue incidence was observed from 1995 to 2002 but must be taken cautiously.
Méndez-Lázaro et al., 2014 [41]	+	Puerto Rico	PCA and bivariate analysis	1995–2009	SLP, MSL, Temperature, Wind, SST, and rainfall	Dengue cases	None	Pearson’s correlation, Mann–Kendall trend test, and logistic regressions	A positive association of precipitation and forested and scrubland habitats with dengue cases was observed.
Buczak et al., 2018 [54]	+		SARIMA and Ensemble models	1990–2009	Rainfall and temperature	Dengue cases		RMSE and MAE	Mixed results for Puerto Rico
Puggioni et al., 2020 [55]	++		Hierarchical Bayesian	1990–2004	Rainfall and temperature	Dengue cases	Spatiotemporal factors	RMSE	
Nova et al., 2021 [42]	++		Empirical Dynamic Modeling	1990–2009	Rainfall and temperature	Dengue cases	Susceptibles index (*λ*)	Pearson’s correlation coefficient and RMSE	Rainfall and susceptibles index were significant drivers of dengue incidence beyond seasonality. However, temperature was not a significant driver beyond seasonality. High host susceptibility allows seasonal climate suitability to fuel large dengue epidemics in San Juan, Puerto Rico.
Depradine and Lovell, 2004 [33]	+	Barbados	Lagged cross-correlation and multiple regression analysis	1995–2000	Rainfall, temperature, and VP	Dengue cases	None	99% CI	VP had the strongest correlation with dengue cases at 6 weeks lag, minimum temperature at 12 weeks lag and max. temperature at 16 weeks lag while there was a negative correlation with wind speed and rainfall with dengue cases.
Parker and Holman, 2014 [51]	++		Logistic model	1992–1996	Rainfall and temperature	Dengue cases	Drought, floods, and storms	CI, SE, and AIC	Mean monthly temperature was the most important factor affecting the duration of both inter-epidemic spells (*β =* 0.543; confidence interval (CI) 0.4954, 0.5906) and epidemic spells (*β =* −0.648; CI −0.7553, −0.5405). Drought conditions increased the time between epidemics. Increased temperature hastened the onset of an epidemic, and during an epidemic, higher mean temperature increased the duration of the epidemic.
Lowe et al., 2018 [37]	++	Barbados	DLNM and Bayesian model	1999–2016	Rainfall and temperature	Dengue cases	None	Area under the curve ROC (AUC)	Low rainfall/drought is positively associated with dengue incidence and increased rainfall after 1–5 months of drought. Failure to predict 2 outbreak peaks of CHIKV and ZIKV.
Douglas et al., 2020 [43]	+		Cross-sectional epidemiology	2008–2016	Seasonality (rainfall)	Dengue cases	Age, sex, and location	95% CI	Peak dengue incidence was observed during the wet season.
Henry and de Assi Mendonça et al., 2020 [56]	+	Jamaica	WADI	1995–2018	Rainfall, temperature, and LST	Dengue cases	WADI Socioeconomic (GIS, urban, and RCP)	SCME	High vulnerability in urban vs. rural areas, expansion to higher latitudes. RCP8.5
Francis et al., 2023 [36]	+	Grenada	Negative binomial regression	2010–2020	Rainfall and temperature	Dengue cases	None	95% CI	In 2013, 2018, and 2020, the driest years, the highest number of DF cases were observed. Other factors may explain these high numbers of DF cases: (1) frequent sporadic heavy rainfall and (2) poor water storage practices in dry season.
Petrone et al., 2021 [32]	++	Dominican Republic	Index P (Bayesian)	2012–2018	Temperature and relative humidity	Dengue cases	R_eff_, AaS scores, CHIKV, and ZIKV	Pearson’s R correlation coefficient	Temperature and humidity analysis (Index P) showed that dengue outbreaks peaked after a period characterized by high transmission potential, just as transmission potential was beginning to wane. Variability in seasonal weather patterns and vectorial capacity did not account for differences in the timing of emerging disease outbreaks.
Bultó et al., 2006 [57]	+	Cuba	EOF	1961–2003	Temperature, rainfall, VP, and relative humidity	Dengue cases	Bultó index, life quality, and degree of poverty	EOF analysis	More frequent outbreaks, changes in seasons and spatial patterns, and less climate variability inland were observed.
Díaz-Quijano and Waldman, 2012 [44]	+	Spanish-speaking Caribbean and Non-Spanish-speaking Caribbean	Poisson regression	1995–2009	Rainfall	Dengue cases	HDI, population density, per capita GEH	95% CI	Rainfall, the sole climatic variable investigated, was associated with dengue mortality (RR = 1.9 [per 10^3^ L/m^2^]; 95% CI = 1.78–2.02) along with population density.
Gharbi et al., 2011 [50]	++	Guadeloupe	SARIMA	2000–2007	Temperature, humidity, and rainfall	Dengue cases	None	RMSE, Wilcoxon signed ranks test, and Pearson’s correlation	Temperature was associated with increased model predictability of dengue incidence forecasting more than rainfall and humidity. Rainfall was not correlated. Minimum temperature at lag 5 weeks was best—RMSE = 0.72.
Limper et al., 2016 [49]	++	Curaçao	DNLM and GAM	1999–2009	Temperature, humidity, and rainfall	Dengue cases	None	95% CI and RR, chi-squared test, and RR	Increases in mean temperature are associated with lower dengue incidence but lower temperatures with higher dengue incidence. Rainfall decreased dengue incidence.
Limper et al., 2010 [34]	+		Non-parametric Spearman’s correlation test	1999–2009	Temperature, rainfall, and humidity	Dengue cases	None	Non-parametric Spearman’s correlation test	
Chadee et al., 2007 [45]	+	Trinidad	Population-based	2002–2004	Rainfall and temperature	Dengue cases	Breteau index	None	Rainfall strongly correlated with dengue disease but no correlation with temperature.
Amarakoon et al., 2008 [48]	++	Trinidad, Barbados, and Jamaica	Correlation analysis including lag	1980–2003	Rainfall and temperature	Dengue cases	MAT, AMAT, A_t_, and D_ot_	Correlation coefficient (*r*)	The yearly patterns of dengue exhibited a well-defined seasonality, with epidemics occurring in the latter half of the year following the onset of rainfall and increasing temperature and a higher probability of epidemics occurring during El Niño periods.
Boston and Kurup, 2017 [46]	+	Guyana	Correlation analysis	2009–2014	Rainfall, temperature, and humidity	Dengue cases	Malaria and leptospirosis	Pearson’s R correlation and correlation coefficient (*r*)	Rainfall strongly associated with dengue incidence but not temperature and humidity.
Ferreira et al., 2014 [58]	+	Guyana, Suriname, Cuba, and multiple Caribbean countries	Correlation analysis	1995–2004	ENSO index	Dengue cases	South Oscillation index (SOI)	Correlation coefficients (*r*)	A higher DF incidence was noted in Cuba, confirming a possible positive ENSO influence.
Gagnon et al., 2001 [47]	+	Suriname and French Guiana	Correlation analysis	1965–1992	Rainfall, temperature, and ENSO cycles	Dengue cases	Monthly river height	Fisher’s exact test, Fisher’s z-transformation, and Quenouille’s method	A statistically significant relationship was observed between El Niño and dengue epidemics in Colombia, French Guiana, Indonesia, and Surinam. The number of DHF cases is highest when a prolonged drought precedes the rainy season.
Adde et al., 2016 [35]	++	French Guiana	Lagged correlation and logistic regression	1991–2013	SST and SLP	Dengue cases	SOI and MEI	Student’s *t* test, Spearman’s lagged correlation, AIC, and AUC	The climatic indices assessed in this study were important for DF monitoring and for predicting outbreaks in French Guiana over a period of 2–3 months. An important rainfall deficit at the end of the dry season enhances the risk of epidemic in the following year.

++ for very high quality, + for high quality and − for low quality; dengue cases refer to laboratory-confirmed dengue infections via NS1 and RT-PCR testing not via syndromic surveillance; Akaike information criteria (AICc); *Aedes aegypti* suitability (AaS) scores; Average Moving Average Temperature (AMAT); analysis of variance (*ANOVA*); Autoregressive Integrated Moving Average (ARIMA); area under the curve ROC (AUC); confidence interval (CI); distributed lag model (DLM); distributed lag nonlinear model (DLNM); Dengue Onset Time (D_ot_); Ecological Niche model (ENM); El Niño Southern Oscillation (ENSO); empiric orthogonal function (EOF) analysis; EVI (Enhanced Vegetation Index); Generalized Additive model (GAM); government expenditure on health (GEH); geographical information system (GIS); generalized linear model (GLM); human development index (HDI); integrated nested Laplace approximation (INLA); land surface temperature (LST); mean absolute error (MAE); Moving Average Temperature (MAT); Tukey’s multiple comparison test (MCT); multivariate ENSO index (MEI); mean sea level (MSL); Principal Component Analysis (PCA); Representative Concentration Pathway 4.5 (RCP4.5); Representative Concentration Pathway 8.5 (RCP 8.5); size of susceptible population (R_eff_); root mean square error (RMSE); relative risk (RR); Seasonal Autoregressive Integrated Moving Average (SARIMA); spatial multi-criteria evaluation (SCME); standard error (SE); South Oscillation index (SOI); sea level pressure (SLP); sea surface temperature (SST); variance inflation factor (VIF); vapor pressure (VP); coefficient of variation *R*^2^; Water-Associated Disease Index (WADI).

Two independent researchers assessed the quality level of selected studies published between 2001 and 2023. An overview of the selected studies is given in Table 2, and the analysis of the metrics used in each study to assess model performance and their respective numerical values for the best and worst candidate models is summarized in Table 3. Ten (10) studies obtained a very high-quality score (++) [35,37,39,40,41,48,49,50,51,55], fifteen (15) received a high-quality score (+) [32,33,34,36,38,39,42,43,44,46,47,53,54,56,57,58], and one (1) study obtained a low-quality score (−) [45] (Table 1 and Table 2). The majority of studies took place in the northern Caribbean—nine (9) had been conducted in Puerto Rico [38,39,40,41,42,52,53,54,55], two (2) studies involving Jamaica [48,56], Cuba [57,58], and Dominican Republic [32,44]. In the southern Caribbean, five (5) studies were conducted involving Barbados [33,37,43,48,51], two (2) studies each were conducted in Curacao [34,49], Trinidad and Tobago [45,48], Guyana [46,58], Suriname [47,58], and French Guiana [35,47]. One study each was conducted in Grenada [36] and Guadeloupe [50]. One study was conducted involving multiple Spanish-speaking and non-Spanish-speaking countries [44]. All selected studies were conducted with various model designs using aggregated data sets. Four (4) studies performed descriptive and Bayesian regression data analyses [32,37,38,55], one (1) study utilized an empirical dynamic model, four (4) studies used correlation analysis [34,47,48,58], four (4) studies used regression analyses [35,36,44,52], and another used multivariate stochastic models [53]. Other studies used SARIMA [50,54], DLM [39] and DLNM models [37,49], PCA and bivariate models [41], wavelet analysis model [40], a logistic model [51], a spatiotemporal model using a Water-Associated Disease Index (WADI) [56], a multimode model using applied empiric orthogonal function (EOF) [57], a cross-sectional epidemiology method [43,59], and a population-based epidemiological study design [45,60,61,62], respectively (Table 2 and Table 3).

The time span of study data ranged from 2 to 28 years, based on monthly, seasonal, or annual climate data with varying time lags (Table 2). After a careful review of the models presented within the selected studies, 21 studies were selected and examined to assess model performances for the best and worst candidate models (Table 3). Evaluation of the best and worst candidate models was not performed in 23.8% (5/21) of this refined list of studies due to the unavailability of suitable data (Table 3).

**Figure 3:**
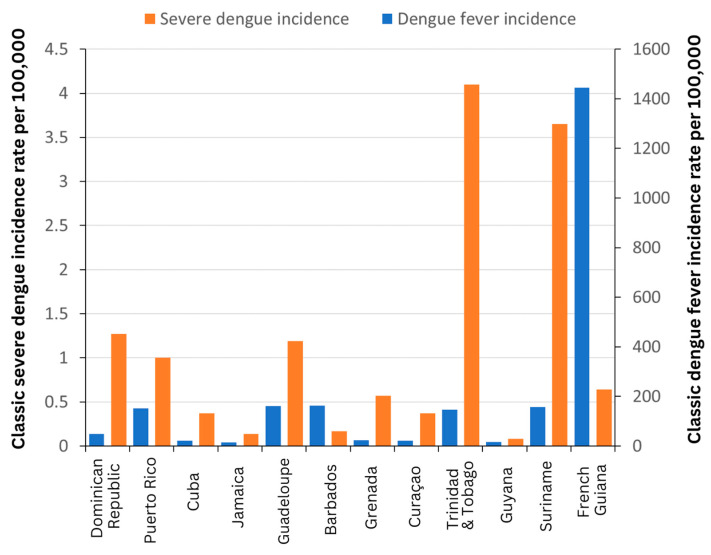
The dengue incidence rates per 100,000 and severe dengue incidence rates per 100,000 from the Caribbean countries. The Caribbean countries included represent those countries where the selected studies in this review were conducted. Countries are grouped into the northern Caribbean grouping (Dominican Republic, Puerto Rico, Cuba, and Jamaica), Eastern/Southeastern Caribbean grouping (Guadeloupe, Barbados, Grenada, and Curaçao), and southern Caribbean grouping (Trinidad and Tobago, Guyana, Suriname, and French Guiana). This data was abstracted from Cafferata et al., 2013 [63].

A comparison of Caribbean countries where the selected studies investigating climate and dengue have been conducted was performed with the relevant dengue and severe dengue incidences from a previous study (Figure 3) [63]. Regarding severe dengue incidence rates, the highest rates were observed among the southern Caribbean grouping (Trinidad and Tobago, Guyana, Suriname, and French Guiana), with Suriname and Trinidad and Tobago reporting the highest rates (Figure 3). The next highest severe dengue incidence rate was reported for the northern Caribbean grouping (Dominican Republic, Puerto Rico, Cuba, and Jamaica), with the Dominican Republic recording the highest rate among this grouping. Finally, Guadeloupe reported the highest rate among the eastern/southeastern Caribbean grouping (Guadeloupe, Barbados, Grenada, and Curaçao). For dengue incidence rates, the southern Caribbean grouping (Trinidad and Tobago, Guyana, Suriname, and French Guiana) reported the highest overall rates, namely French Guiana and Suriname (Figure 3). During ENSO events (La Niña and El Niño), generally, there are opposing conditions found in the northern Caribbean compared to the southern Caribbean. During El Niño, generally, the northern Caribbean experiences higher rainfall while the southern Caribbean experiences drier conditions, and during La Niña, the northern Caribbean experiences drier conditions and the southern Caribbean higher rainfall.

**Table 3:** Analysis of selected published peer-reviewed studies and the relevant metrics used to assess model performance and their respective numerical values for the best and the worst candidate models.

Study	Quality	Study Location	Study Design	Statistical Methods	Metric	Best Model Value	Worst Model Value
Keating et al., 2001 [52]	++	Puerto Rico	Multivariate linear regression	Regression analysis and Durbin–Watson test	R-squared	0.71	0.62
					F-value	49.94	67.22
					SE	102.8	116.9
Schreiber et al., 2001 [53]	++		Multivariate stochastic models	Pearson’s product–moment correlation coefficient	Adjusted R-squared	0.88	0.14
Jury, 2008 [38]	+					0.02	0.0001
Johansson et al., 2009a [39]	+		Poisson regression models		N/A	N/A	N/A
Johansson et al., 2009b [40]	+		Wavelet analysis	Monte Carlo (MC) significance	MC significance	0.006	0.006
Méndez-Lárazo et al., 2014 [41]	+		Principal Component Analysis	Logistic regression	*p*-value	N/A	N/A
			Pearson correlation coefficient				
			Mann–Kendall trend test				
			Logistic regression				
Buczak et al., 2018 [54]	++		SARIMA and Ensemble models	Time series methods	Log loss	−1.8	−6.4
Puggioni et al., 2020 [55]	++		Hierarchical Bayesian		Mean squared error	8.632	14,055.18
					Mean absolute percentage error	2.663	372.01
					Mean absolute error	2.228	86.56
					Relative bias	−0.001	0.49
					Relative mean separation	0.436	0.97
					Root mean squared error	2.951	118.11
Nova et al., 2021 [42]	++		Empirical Dynamic Modeling	Correlation analysis	Pearson’s correlation coefficient	0.9697	0.38
					Root mean squared error	37.14	57.34
Depradine and Lovell, 2004 [33]	+	Barbados					
			Lagged cross-correlation and multiple regression analysis	Correlation analysis	Pearson’s correlation coefficient	0.7	0.25
Parker and Holman, 2014 [51]	++		Logistic model	Akaike information criterion (AIC) and 99% CI	N/A (no comparison made)	N/A	N/A
					Standard error	0.0001	172.4
Lowe et al., 2018 [37]	++		DLNM and Bayesian model	Area under the curve ROC (AUC)	AUC	0.90	0.75
				Likelihood ratio R-squared	*R*^2^_LR	0.68	0.23
				Deviance information criterion	DIC	1664.94	1801.36
		Barbados					
Douglas et al., 2020 [43]	+		Cross-sectional epidemiology	95% CI	CI	N/A	N/A
Henry and de Assi Mendonça et al., 2020 [56]	+	Jamaica	Water-Associated Disease Index	Color-coded vulnerability	N/A	N/A	N/A
Amarakoon et al., 2008 [48]	+		Time series analysis	Correlation analysis	Pearson’s correlation coefficient	>0.71	N/A
Francis et al., 2023 [36]	+	Grenada	Negative binomial regression	95% CI	CI	N/A	N/A
Díaz-Quijano and Waldman, 2012 [44]	++	Cuba	Poisson regression	Correlation coefficient, rate ratio, CI	Pseudo *R*^2^	49.8%	48.3%
Bultó et al., 2006 [57]	++		Statistical variability analysis	Bultó index	IB index	18.77	1109
Boston and Kurup, 2017 [46]	+	Guyana	Correlation and regression analysis	Correlation coefficient	r	0.7	0.1
Ferreira et al., 2014 [58]	+	Guyana, Belize, Suriname, Cuba, and multiple Caribbean countries	Frequency analysis	Annual dengue frequency	Mean annual frequency	18.27	N/A
Adde et al., 2016 [35]	++	French Guiana	Logistic binomial regression model	Akaike information criterion (AIC)	AIC	27	31
				Area under curve (AUC)	AUC	0.88	0.75
				Standard error	SE	0.02	1.42

++ as very high quality, + as high quality, and − as low quality; dengue cases refer to laboratory-confirmed dengue infections via NS1 and RT-PCR testing not via syndromic surveillance; Akaike information criteria (AICc); *Aedes aegypti* suitability (AaS) scores; Average Moving Average Temperature (AMAT); analysis of variance (*ANOVA*); Autoregressive Integrated Moving Average (ARIMA); area under the curve ROC (AUC); confidence interval (CI); distributed lag model (DLM); distributed lag nonlinear model (DLNM); Dengue Onset Time (D_ot_); Ecological Niche model (ENM); El Niño Southern Oscillation (ENSO); empiric orthogonal function (EOF) analysis; EVI (Enhanced Vegetation Index); Generalized Additive model (GAM); government expenditure on health (GEH); geographical information system (GIS); generalized linear model (GLM); human development index (HDI); integrated nested Laplace approximation (INLA); land surface temperature (LST); mean absolute error (MAE); Moving Average Temperature (MAT); Tukey’s multiple comparison test (MCT); multivariate ENSO index (MEI); mean sea level (MSL); Principal Component Analysis (PCA); Representative Concentration Pathway 4.5 (RCP4.5); Representative Concentration Pathway 8.5 (RCP 8.5); size of susceptible population (R_eff_); root mean square error (RMSE); relative risk (RR); Seasonal Autoregressive Integrated Moving Average (SARIMA); spatial multi-criteria evaluation (SCME); sea level pressure (SLP); sea surface temperature (SST); variance inflation factor (VIF); coefficient of variation *R*^2^; Water-Associated Disease Index (WADI).

## 4. Discussion

Our goal was to investigate the relationships between human DENV infection rates and short- and medium-term climate variability in the English- and non-English-speaking wider Caribbean. A number of variables, including biotic, abiotic, geospatial, and climatic ones, were linked to higher chances of human DENV infection.

### 4.1. Rainfall or Precipitation Factor

This was the common climatic variable consistently associated with increased DENV infection risk in the English-speaking and non-English-speaking Caribbean among the selected studies. This climatic factor was associated with most of the selected studies (51.9%) with a variable lag period of up to 3 months, which could be related to the influence of rainfall on creating ideal conditions for sustained vector population growth, including vegetative growth and water for oviposition and larval maturation. Studies in other regions also support the positive association of rainfall/precipitation with dengue infection risk, including the Caribbean, Southeast Asia, and South America [64,65,66]. Conversely, rainfall has been negatively associated with dengue incidence in several countries in this study [34,35,36,37] and is supported by a study in Singapore [67]. Rainfall patterns in the Caribbean are characterized by a dry season (December–May) and a wet season (June–November). The El Niño Southern Oscillation (ENSO), which is associated with a sea surface temperature (SST) gradient anomaly between the eastern Tropical Pacific Ocean and the Caribbean Sea, directly affects this rainfall distribution [68]. El Niño is associated with warmer SST in the central and eastern tropical Pacific Ocean, which has been linked to notable drought events in the Caribbean [69]. Conversely, La Niña coincides with below-average SST, leading to wet episodes with historical events occurring in 2010–2011 and 2011–2012. El Niño events in 1997–98 and 2009–2010 have been associated with dengue epidemics in Barbados and other Caribbean countries [48]. The link between El Niño events and dengue epidemics has also been observed in other regions [70,71], thus underscoring the links of dengue epidemics with climate. Extended wet periods can result in flooding with appreciable frequency in the Caribbean due to the severe weather system activity. Flooding can impact mosquito distribution by flushing immature stages of mosquitoes from the environment and potentially reducing mosquito densities [67]. This can then reduce the mosquito–human contact frequency and time duration.

### 4.2. Temperature Factor

Another climatic factor found to positively influence (40.7% of selected studies) human DENV infections in the Caribbean is temperature. Several studies have shown an association between increased temperatures and dengue disease [72]. Temperature can influence mosquito vector growth, population abundance, and dengue disease risk as it promotes vegetation growth, reproduction, and survival rates of mosquito vectors. Specifically, temperature influences both the development rate of and DENV replication rate in mosquito vectors [73]. The average development cycle of mosquitoes ranges from 8 to 10 days from egg to adult mosquito [73]. Female mosquitoes acquire DENV infection during feeding and obtain a bloodmeal from infectious individuals (viremic), after which they can transmit DENV infection during the next 7–14 days or for as long as the female mosquito lives in some cases up to 5 months, provided, they have a sufficient food supply [74]. The extrinsic incubation period (EIP) of DENV, the time taken from a female mosquito ingesting a DENV-infected blood meal to the presence of DENV in salivary glands, is temperature-dependent with shorter EIPs observed at elevated temperatures [74]. Warming temperatures within the Caribbean region will include ocean and land [18]. Excessive warming and humidity can alter human behaviors, promoting behaviors that seek cooling relief, including spending time outdoors, wearing less clothing, opening windows and doors, and bathing in water bodies [75]. This exposes human skin and humans to increased mosquito bites and dengue virus transmission risks.

### 4.3. Multiple Co-Factors

Among select research studies conducted in Caribbean and CARICOM territories, spatial, social, abiotic, and biotic characteristics such as location, land use management, poverty, landcover type, and human development index (HDI) have been linked to human DENV infections.

#### 4.3.1. Mosquito Vector Distribution

Climate variability will impact Neotropical mosquito vectors, their distribution, and activity in the Caribbean. In the Caribbean, the mosquito vectors primarily involved in dengue transmission are *Aedes aegypti* and *Aedes albopictus*. However, given the feeding plasticity of mosquitoes and the observance of DENV infection in *Culex* spp. in Brazil [76], it would not be unexpected if the range of mosquito species infected by DENV is more expansive within the Caribbean. These mosquito vectors often inhabit peridomestic and intra-domestic ecosystems in the Caribbean, including homes, equatorial and tropical forests, swamps, savannas, water storage containers, septic tanks, underground stormwater drainage systems, and salt marshes [17]. Mosquito vectors can adapt to climatic changes, e.g., in drought conditions, mosquitoes may seek to breed in limited available water in underground septic tanks or stormwater drains [77]. Mosquito species diversity, distribution, and abundance can thus be influenced by infrastructure.

#### 4.3.2. Human and Social Factors

Human and social factors contribute to human DENV infection risk [78,79]. Thus, the inclusion of these factors, such as HDI, human population size, and air passenger flux, should be carefully examined to understand human DENV infections and transmission [80,81].

#### 4.3.3. Infrastructure

The presence of man-made infrastructures can influence vector-borne infectious disease dynamics, including underground storm drainage, sewerage networks, and household and commercial septic tanks, by promoting the growth of mosquito vectors [82]. Mosquitoes can breed in small volumes of water and do not require rainwater. Thus, while precipitation levels may be low or absent, the influence of leaking municipal potable water distribution systems can provide a stagnant water source, fostering mosquito breeding. This leak inefficiency is a feature of aging water distribution systems common within the Latin America and Caribbean (LAC) region [83]. Thus, such a negative association of rainfall with human DENV infections should be carefully assessed, and the role of leaking water infrastructure should be examined through geospatial analysis. This would permit analysis of its impact on drainage, mosquito vector distribution, vector population densities, and mosquito-borne diseases.

Solid waste management can influence human DENV infections by controlling the number of breeding sites and population sizes of *Aedes aegypti* mosquitoes [84]. Adequate waste management systems and community participation in source reduction efforts are essential to reduce mosquito breeding sites and control DENV transmission. Solid waste present in the environment due to littering or dumping can increase flood risk, as solid waste can block drainage systems [85]. Future dengue studies should incorporate waste management in risk analysis to understand its influence, especially in population-dense areas. The role of infrastructure in human DENV infections requires more profound research to augment sustainable development in the region.

#### 4.3.4. Travel and Trade

International travel poses a risk for the movement of DENV-infected persons from one region to another. DENV infections among travelers from the U.S. and Canada [86] to the Caribbean have been previously documented. These all point to the risk of importation and exportation of active human dengue infections to and from areas where dengue transmission is endemic. One study of DENV viral genetics in Barbados has hinted at the potential role of travel in introducing epidemic DENV strains from SE Asia to the Caribbean [43]. Another study emphasized the necessity of monitoring sick travelers to offer a snapshot of local introductions and transmission in places with low local surveillance, implying that the recent DENV-3 introductions may constitute a significant public health danger in the area [87]. Furthermore, another Caribbean study indicated that the spread of DENV-1, -2, and -3 in the Americas was linked to airplane traffic, but specific predictors varied for each DENV serotype [81]. Due to the speed and frequency of global travel, dengue–climate risk models should factor in this potential entry risk of new dengue virus strains into endemic regions like the Caribbean. Aircraft wastewater surveillance offers potential for supporting efforts to monitor this risk, as several studies indicate the ability to detect arboviral RNA from wastewater samples even with low caseloads [88,89,90,91]. It complements human and mosquito surveillance and, as a non-intrusive approach, is especially useful for monitoring diseases like dengue and Zika fever, which can be characterized by asymptomatic/silent or mild infections [92].

### 4.4. Limitations

There are limitations noted in the selected studies, including the resolution of climatic variables (typically monthly or weekly), low dengue case numbers, short duration of study, source of data (climate databases rather than local climatic datasets), partial absence of mosquito population, mosquito infection rates and dengue infection dynamics (immunological history) data, waste management data, water infrastructure leak distribution data, absence of other specific climatic/abiotic variables such as wind speed, sea surface temperature (SST) and ENSO. These could all impact the accuracy of predictive models for human dengue infections.

The limitations of this review include the bibliographic search, which is limited by the existing peer-reviewed studies present on the databases searched, namely PubMed and Web of Science. Other pertinent studies not currently indexed at the time of conducting the bibliographic search may exist, so the list of selected may not be all-inclusive.

## 5. Conclusions

Climatic factors, namely rainfall or precipitation, humidity, and temperatures, have been linked to human DF cases in the Caribbean and CARICOM countries. Rainfall and temperature are the climate factors most often positively associated with human DENV infections in these regions. However, more research, including other co-factors influencing DENV entry and spread, such as trade, travel, infrastructure, and humans, would aid in refining the predictive power of climate–DF models. As this region ebbs closer to climate departure, there is a greater need to comprehend the interactions of abiotic and biotic factors in DENV infection risks to achieve better modeling of human infections and outbreak risk assessments.

This systematic review was registered with PROSPERO under the registration number CRD42021290450 on 12 December 2021.

## Figures and Tables

**Table 1:** PECOS criteria followed in the review.

Parameters	Inclusion Criteria	Exclusion Criteria
Population	People with acute DENV infection or DF/NSD/SD/DHF/DSS	Only mosquito DENV infections
Exposure	At least one climate factor	No climate factors
Comparators	N/A	
Outcomes	Dengue case/infection/risk/incidence	Mosquito density/distribution
Study design	Observational, epidemiological, retrospective, predictive modeling study design	Prospective study design

N/A—not applicable; PECOS—patient, intervention/exposure, comparator, outcomes, study design; dengue virus (DENV); dengue fever (DF); non-severe dengue (NSD); severe dengue (SD); dengue hemorrhagic fever (DHF); and dengue shock syndrome (DSS).

## Data Availability

Not applicable.

## Supplementary Materials

The following supporting information can be downloaded at https://www.mdpi.com/article/10.3390/pathogens13090756/s1, Table S1: Scoring system for quality assessment of selected studies.

## Appendix A

The appendix is a summary of the checklist of the relevant aspects of each study under review for the assessment of several quality criteria to permit the scoring of a grade. Please see also Table S1.

**Checklist for Quality Assessment of Selected Studies in the Systematic Review**

A. **Validity of Study Results**
  1. Was a clear focus issue addressed?—Yes/Unsure/No
    Follow on and ask some of these other questions to evaluate the rating.
  2. Which study design was utilized?
  3. Was the appropriate method used to answer the question posed?
  4. Was/Were the climate exposure factor(s) accurately measured to minimize bias?
  5. Was the outcome accurately measured to minimize bias?
  6. Were all the important confounding factors identified?
  7. Are these confounding factors addressed in the design and or analysis?
B. **What are the Study Results?**
  1. What are the results of this study?—Yes/Unsure/No
    Follow and ask some of these other questions to evaluate the rating.
  2. What is the level of precision of the results? What is the level of precision for the risk estimate used?
  3. Are the results believable based on statistics?
  4. How was this study funded?
C. **Applicability of Study Results to This Systematic Review**
  1. Does this study answer the question posed by this systematic review?—Yes/Unsure/No
    Follow on and ask some of these other questions to evaluate the rating.
  2. What is the level of precision of the results? What is the level of precision for the risk estimate used?
  3. Are the results believable?
