# Supplementary material for: The Impact of Climate on Human Dengue Infections in the Caribbean
Source: Pathogens. 2024 Sep 3;13(9):756. doi: 10.3390/pathogens13090756 (PMC11434940; doi:10.3390/pathogens13090756)
Supplement: Supplementary file 1 [file pathogens-13-00756-s001.zip › pathogens-2984975-supplementary.pdf]

## Table S1. Scoring system for quality assessment of selected studies

| Criteria | - | + | ++ |
|---|---|---|---|
| **Selection** | No description | Partial description | Full description |
| **Study design** | No description | Partial description | Full description |
| **Risk of bias** | No description of bias reduction | Partial description of bias reduction | Full description of bias reduction |
| **Data collection** | No description/reliable source | Partial description | Full description & reliable source |
| **Observational time period** | Less than a single year | Single year | Greater than 1 year |
| **Analysis** | Non-appropriate method for study design (correlation) | Not a fully appropriate method for study design (linear regression) | Appropriate method for study design (Poisson regression) |
| **Interpretation of factors** | No statistical test or measure | Statistically significant but with no reported measure | Statistically significant with measures |
| **Funding of study** | No description | Partial description | Full description |
| **Applicability to review study question** | Not applicable | Partially applicable | Applicable |
| **Dengue diagnosis** | No description | Partial description/suspected cases | Full description/laboratory confirmed cases |

**Grading:**

++ for very high quality studies scoring more than 7 (++)

\+ for high quality studies scoring at least 7 (++)

\- for low quality studies scoring less than 7 (++)
